# Editorial: Advances in biotechnological approaches for reproductive tissue engineering

**DOI:** 10.3389/fbioe.2025.1738886

**Published:** 2025-11-27

**Authors:** Gustavo Henrique Doná Rodrigues Almeida, Fabiana Fernandes Bressan, Maria Angelica Miglino, Moon Suk Kim, Ana Claudia Oliveira Carreira

**Affiliations:** 1 Graduate Program in Anatomy of Domestic and Wild Animals, Department of Surgery, Faculty of Veterinary Medicine and Animal Science, University of São Paulo, São Paulo, Brazil; 2 Laboratory of Development and Innovation, Butantan Institute, São Paulo, Brazil; 3 Department of Veterinary Medicine, Faculty of Animal Science and Food Engineering, University of São Paulo, São Paulo, Brazil; 4 Graduate Program in Structural and Functional Interactions in Rehabilitation, School of Medicine, Universidade de Marília, Marília, Brazil; 5 Graduate Program in Animal Health, Production and Environment, School of Veterinary Medicine, Universidade de Marília, Marília, Brazil; 6 Department of Molecular Science and Technology, Ajou University, Suwon, Republic of Korea; 7 Center for Natural and Human Sciences, Federal University of ABC, Santo André, Brazil

**Keywords:** reproduction, biomaterials, tissue engineering, regenerative medicine, reproductive tissues

## Introduction

The development of artificial grafts capable of recapitulating native tissue microarchitecture represents one of the most ambitious challenges in regenerative medicine. Such constructs provide not only a framework to investigate molecular and cellular mechanisms in *in vitro* microenvironments but also a foundation for restoring or reestablishing tissue homeostasis *in vivo*. In this context, tissue-engineering strategies that integrate multiple cell types, biomaterials of diverse origins, and bioactive signaling cues have emerged as powerful tools to mimic the morphophysiological complexity of various organs and systems. More recently, these approaches have been extended to reproductive tissues, aiming to restore fertility, elucidate the underlying mechanisms of gestational loss and gamete dysfunction, and enhance the success rates of assisted reproduction. The creation of biomimetic microenvironments capable of supporting oocyte maturation, embryo development, and tissue repair is among the most promising frontiers in reproductive bioengineering.

Unlike other organs, male and female reproductive tissues exhibit remarkable plasticity and dynamic remodeling driven by cyclical hormonal fluctuations. This intrinsic variability makes their structural and biochemical microenvironments particularly challenging to reproduce *in vitro* and highly sensitive to environmental disturbances. As a result, several mechanisms associated with infertility and gestational disorders remain incompletely understood. Furthermore, due to their complex histoarchitecture, these tissues often undergo inefficient healing, which may result in fibrosis or degeneration and compromise reproductive function.

To overcome these challenges, innovative strategies are required to improve tissue regeneration and to develop new methodologies for the generation, maturation, and culture of gametes and embryos under controlled conditions. Recent advances include the use of stem cells, tissue-derived biomaterials, dynamic culture systems, and artificial microphysiological platforms. Nevertheless, reproductive tissue engineering remains in its early stages, demanding refined and standardized methodologies to accelerate translation. In a global scenario marked by declining fertility rates and the demand for sustainable livestock production, such biotechnological innovations will be essential to secure both reproductive health and food security in the coming decades.

## Extracellular matrix-derived scaffolds and biomaterials

Decellularized matrices derived from reproductive and placental tissues have shown remarkable translational potential in both tissue engineering and regenerative medicine, serving as dynamic platforms that support cellular differentiation and promote tissue remodeling. In this context, Almeida et al. explore the use of acellular porcine placental membranes as a biomaterial for tissue repair, revealing how their native matrix composition can guide cell migration and epithelialization. The study emphasizes that, beyond serving as structural scaffolds, placental ECMs retain signaling molecules that modulate inflammation and healing, reflecting the unique immunotolerant and angiogenic properties of fetal tissues. This approach highlights the placenta not only as a biological interface essential for development, but also as a scalable and ethically accessible material for translational bioengineering.

At the organ level, Di Filippo et al. present bovine decellularized testicular scaffolds as a promising 3D platform for testis bioengineering. Through a comparative analysis of three decellularization protocols followed by recellularization with primary testicular cells, the authors demonstrate that maintaining the native ECM microarchitecture is essential for proper cell adhesion, spatial organization, and the recreation of a germ-cell niche. These results represent a key step toward developing *ex vivo* models of spermatogenesis and future strategies for fertility restoration. Importantly, the study underscores the delicate balance between efficient DNA removal and structural preservation, reinforcing the value of standardized quantitative criteria, such as residual DNA content, glycosaminoglycan and collagen retention, and mechanical stability, to guide the optimization of scaffolds for reproductive tissue applications.

Although developed from thyroid tissue, the study by Zhang et al. offers an elegant proof of concept for injectable, tissue-specific ECM hydrogels with preserved ultrastructure and excellent biocompatibility. Beyond its endocrine origin, this work provides a methodological blueprint readily adaptable to reproductive tissues, where injectable and in situ–gelling matrices could be transformative. Such systems hold particular promise for endometrial and ovarian regeneration, as well as for creating biomimetic microenvironments that sustain oocyte maturation, embryo development, and implantation. By bridging decellularization chemistry, hydrogel formulation, and biophysical characterization, the study expands the technological horizon of ECM-based biomaterials, reinforcing their potential to reproduce the dynamic, hormonally responsive niches that define reproductive tissue physiology.

Finally, Kang and Yang outline the translational roadmap for decellularized vascular grafts, emphasizing key challenges such as re-endothelialization, antithrombogenic modulation, and peri-implant remodeling that must be addressed to achieve long-term functionality. Although focused on vascular systems, their discussion is deeply relevant to reproductive tissue bioengineering, where insufficient vascularization remains a major limitation. Concepts such as perfusion decellularization, luminal endothelialization, and biomechanical preconditioning are equally critical for the development of durable uterine and ovarian grafts, as well as for placenta-inspired constructs requiring efficient nutrient and oxygen exchange. Integrating these vessel-oriented principles into the design of reproductive ECM scaffolds could thus represent a decisive step toward achieving physiological integration and clinical translation in reproductive regenerative medicine.

## *In vitro* cell models and organoid-based approaches for reproductive engineering

Reproductive tissue engineering increasingly relied on 3D cell culture systems, organoids, and bioprinting to reproduce the structural and functional complexity of the endometrium and myometrium. These advanced models not only deepen our understanding of hormonal regulation and implantation dynamics but also open avenues for testing regenerative therapies and improving assisted reproduction outcomes. Ma et al. present a remarkable study demonstrating that self-renewing endometrial epithelial organoids can successfully engraft and differentiate into functional glands when transplanted into injured endometrium. Their findings reveal the intrinsic capacity of organoid-derived epithelial cells to restore glandular structure and secretory function, highlighting organoid technology as a powerful regenerative strategy for uterine repair and infertility associated with endometrial damage.

Ma et al. presented a remarkable study demonstrating that self-renewing endometrial epithelial organoids can successfully engraft and differentiate into functional glands when transplanted into injured endometrium. Their findings reveal the intrinsic capacity of organoid-derived epithelial cells to restore glandular structure and secretory function, highlighting organoid technology as a powerful regenerative strategy for uterine repair and infertility associated with endometrial damage.

Complementing this approach, Ulrich et al. developed a bioprinted model of the pregnant human uterine myometrium, combining smooth muscle cells and extracellular matrix components to reproduce tissue contractility and mechanical responsiveness. This model offers an unprecedented tool to investigate uterine physiology during pregnancy, including the mechanisms governing contraction, stretch response, and preterm labor.

At the interface between implantation biology and tissue modeling, Pennarossa et al. explored bioengineered constructs to reproduce mammalian implantation *in vitro*. By establishing an artificial environment that enables trophoblast-endometrium interaction, their study provides a foundation for dissecting early implantation events, embryo–maternal communication, and the molecular factors that determine implantation success or failure.

Following this methodological pathway, Agustina-Hernández et al. reviewed recent biotechnological advances in human endometrial modeling, tracing the evolution from traditional 2D culture toward sophisticated 3D and organoid systems. Their analysis emphasizes how dynamic culture conditions, hormonal cues, and matrix composition influence endometrial behavior, offering insights for more physiologically relevant *in vitro* models.

## Reproductive tissue engineering for fertility and regeneration

Biotechnological approaches focused on the germ-cell microenvironment, along with improved techniques for their isolation and preservation, represent a fundamental pillar of reproductive bioengineering. *In vitro* technologies play a crucial role in supporting gamete maturation and viability, forming the basis for fertility restoration and for modeling germline dysfunctions.

In this context, Pasquariello et al. presented a detailed protocol for the isolation, identification, and quality assessment of bovine spermatids, followed by the evaluation of their fertilizing potential *in vitro*. Their work establishes a reliable methodological foundation for studying spermatogenesis and developing assisted reproductive techniques in large-animal models. By highlighting the delicate balance between purity, viability, and differentiation potential of germ cells, this study provides valuable insights for both animal reproduction and future applications in human fertility preservation.

In a broader translational perspective, Kim et al. proposed the non-human primate as a next-generation model for female reproductive engineering. Their review underscores the physiological and anatomical parallels between primate and human reproductive systems, offering a realistic platform to test regenerative and biotechnological interventions. From ovarian folliculogenesis to uterine regeneration, the non-human primate model emerges as a critical link between experimental findings and clinical practice.

## Cross-disciplinary innovations and preservation technologies

While most advances in reproductive tissue engineering have traditionally focused on organ- or cell-specific strategies, the field is now being reshaped by a wave of cross-disciplinary innovations emerging from broader areas of regenerative medicine, materials science, and cryobiology. Developments originally aimed at improving tissue preservation, drug delivery, or scaffold design in other organ systems are increasingly providing conceptual and technological frameworks that can be directly adapted to reproductive applications. These include the refinement of biomaterial responsiveness, advances in temperature-controlled storage, and novel biofabrication and preservation methodologies that can sustain cell viability and tissue function over extended periods.

Gokaltun et al. introduced a supercooled preservation system for primary hepatocyte monolayers, demonstrating that controlled hypothermic conditions can maintain cell viability and metabolic function without cryoprotectants. Although developed for hepatic tissue, this approach offers important implications for reproductive cell and tissue preservation, particularly for oocytes, embryos, and engineered endometrial or ovarian constructs, where maintaining structural and functional integrity during storage and transport remains a critical challenge.

In parallel, Wu et al. provided a comprehensive overview of smart, stimuli-responsive hydrogels and their potential in bone tissue engineering, describing how temperature, pH, and enzymatic responsiveness can be harnessed to modulate degradation and drug release. The principles underlying these adaptable materials are equally relevant to reproductive systems, where dynamic hormonal and biochemical changes require biomaterials capable of responding to local cues. Such intelligent systems could enable the development of next-generation ECM-based scaffolds that adapt to cyclical environments, supporting tissue regeneration and embryo implantation.

## Conclusion

Reproductive tissue engineering is emerging as a cutting-edge field in regenerative medicine, uniting advances in biomaterials, cell biology, and biofabrication to tackle the inherent complexity of reproductive organs. The integration of decellularized extracellular matrices, 3D organoid systems, and germ-cell engineering has enabled the reconstruction of key structural and functional features of endometrial, ovarian, and testicular tissues under controlled conditions. These strategies are not only deepening the understanding of gametogenesis, implantation, and tissue remodeling, but also providing clinically relevant models to study infertility and reproductive pathologies. Despite these advances, major challenges remain, particularly the need for standardized protocols, effective vascularization, and functional integration of engineered tissues to ensure reproducibility and translational reliability. Progress in this field will depend on sustained collaboration among experts in materials science, reproductive biology, and clinical research, driving the translation of experimental achievements into safe, effective, and scalable therapies for both human and animal reproductive health.

